# Enhancing child dietary diversity through cooking demonstration and nutritional education in rural Lao PDR

**DOI:** 10.1186/s41182-023-00571-3

**Published:** 2024-01-08

**Authors:** Yu Sato, Somboun Khamphithoun, Khamphanavanh Saiyachak, Hisao Ando, Takaaki Ishizuka, Shinjiro Saeki, Miki Miyoshi

**Affiliations:** 1 Our Lady of the Snow St. Mary’s Hospital, Tsubuku Honmachi 422, Kurume, Fukuoka Japan; 2 Non-Profit Organization ISAPH Tokyo Office, OWK Bld. 3F, Shimbashi 3-5-2, Minato-Ku, Tokyo, Japan; 3 Aomori University of Health and Welfare, Oaza-Hamatate Mase 58-1, Aomori, Aomori Japan; 4 Khammouane Provincial Health Department, Thakhek, Khammouane Lao PDR; 5 Non-Profit Organization Shockonken, Tokyo, Japan

**Keywords:** Dietary diversity, Lao PDR, Children under five, Nutrition, Food security

## Abstract

**Background:**

Efforts to prevent malnutrition in children under five are crucial for both short-term and long-term impact, especially in resource-constrained low- and middle-income countries, where ensuring minimal food diversity remains an urgent challenge. Our organization implemented initiatives to improve dietary diversity among children under five in rural areas of Lao People’s Democratic Republic (Lao PDR).

**Methods:**

We carried out educational and awareness programs directed at caregivers of children aged 6–59 months. These programs were delivered by healthcare professionals and trained community volunteers in specific areas of Xaybouathong District, Khammouane Province. To evaluate the impact of our interventions, we conducted surveys both at the beginning and end of the project. We designated the Individual Dietary Diversity Score IDDS as the objective variable, serving as an indicator of child dietary diversity. Using sociodemographic and economic indicators as explanatory variables, we assessed the impact of the intervention through multivariate analysis with a generalized linear model as well as a bivariate analysis.

**Results:**

The comparison between 210 children at baseline and 205 children at endline revealed a significant increase in IDDS among children aged 6–23 months (from 3.36 to 4.22) and children aged 24–59 months (from 3.29 to 3.83). Multivariate analysis indicated a significant association between the intervention effect (baseline vs. endline) and the village of residence. Furthermore, significant improvements were observed in each food group that constitute IDDS, including vegetables and fruits, eggs, and legumes and nuts.

**Conclusions:**

Even in resource-limited settings, such as rural areas of Lao PDR, it is possible to improve child dietary diversity through educational approaches that encourage the utilization of locally available foods.

## Background

Infant and child malnutrition has short-term effects such as diseases and mortality and increases the risk of future Non-Communicable Diseases (NCDs), thereby hindering lifelong human and societal development [1]. Typical indicators used to measure malnutrition in low- and middle-income countries (LMICs) include stunting and wasting among children under 5 years of age [2]. As of 2023, it is estimated that 1.48 million children under five worldwide suffer from stunting, and 450,000 children suffer from wasting, with a significant concentration in Southeast Asia and Africa [3]. Stunting represents chronic malnutrition and is assessed by the difference between the average height for a given age and the child’s actual height. It is associated with chronic infections, micronutrient deficiencies, and inadequate intake of essential nutrients in a balanced manner [4].

One evidence-based approach to preventing stunting among children under five is ensuring dietary diversity [5, 6]. Dietary diversity involves categorizing foods into various food groups to ensure a balanced intake of nutrients required by pregnant and lactating mothers and children under 5. In LMICs, where a detailed assessment of nutrient intake may be challenging, dietary diversity is used as an indicator to measure the minimal diversity of foods consumed by infants and pregnant women [7, 8].

Factors threatening dietary diversity often relate to food security issues, such as insufficient quantity of food or difficulties in physical and economic access to food. However, previous reports have also shown that even when sufficient and accessible food is available, individuals may consume only a limited variety of foods for various reasons [9]. As the availability and accessibility of food vary across different regions, it is essential to investigate the presence and consumption of food in each area to ensure dietary diversity.

Lao People’s Democratic Republic (Lao PDR) is one of the LMICs in Southeast Asia, and its under-five mortality rate is high at 33 per 1000 live births compared to neighboring countries. Stunting affects around 21.5% of children under 5 years in urban areas, with a significant prevalence observed at 37.2% in rural areas. According to the Lao Social Indicator Survey, dietary diversity among children in rural areas is limited, with only 45.3% of children meeting the Minimum Dietary Diversity (MDD) [10]. MDD is the percentage of children 6–23 months of age who consumed foods and beverages from at least five out of eight defined food groups during the previous day.

In rural areas, there are various challenges compared to urban areas, such as lower literacy rates and difficulties in accessing education and healthcare services, which also threaten dietary diversity among children under five in rural Lao [11]). These days, acquiring accurate information to protect one’s health is an alternative; however, in rural areas, it has been reported that reliance on interpersonal communication is much more common [12]. Therefore, strengthening face-to-face activities could contribute to improving dietary diversity. Against this backdrop, the Our Lady of the Snow St. Maria Hospital and the Non-profit Organization ISAPH collaborated with the Lao health administration to implement activities to improve the dietary diversity of children under five in rural Lao.

The purpose of this study is to measure the effectiveness of the activity conducted to enhance dietary diversity among children and to report on the actual situation and changes in the diversity of children’s diets.

## Methods

### Study site

Khammouane Province, located in the central region of Lao PDR, approximately 330 km southeast of the capital Vientiane, consists of ten districts. Xaybouathong District, situated in the central-southern part, is a small rural area with a population of about 27,000. The district is covered by 40 villages, served by one district hospital and five health centers, where residents can receive primary healthcare services. As this study focused on providing health/nutritional education, three villages were selected from the 20 villages under catchment areas of the district hospital based on their high nutritional improvement needs for children and relatively feasible to access. The chosen villages, Pharkhong, Phakouay-thong, and Phakouay-dong, are each located 7 km to 10 km from the district hospital. However, according to information from the Xaybouathong health administration officials, villages are adjacent to one another, exhibiting no ethnic differences (“Phu-Thai” ethnic group only), and sharing similar environmental backgrounds. Therefore, we recognizing them as having the same geographical and ethnical conditions in this study.

### Health/nutritional education and implementation

The activities were carried out from April 2017 to March 2020, and they were aimed at all mothers and children under the age of five living in Pharkhong, Phakouay-thong, and Phakouay-dong villages. Once a month, healthcare practitioners and community volunteers provided health/nutrition education during the maternal and child health outreach program. The practitioners were either staff from Xaybouathong District Health Office or the district hospital. They were given educational materials and funding to conduct monthly sessions for raising awareness about health and nutrition, as per Lao PDR’s integrated strategy for maternal and child health outreach services [13]. During the period from August 2017 to January 2020, a total of 30 sessions were conducted as part of the project’s intervention activities, with a specific focus on health/nutritional education.

Regarding the community volunteers, there was a need for proper training in health/nutrition education. Hence, members of the Lao Women’s Union were preferential recruited as volunteers, and they underwent 4 months of training. The Lao Women’s Union operates independently from the maternal and child health services of the public health administration within villages [14]. With the aim of ensuring the sustainability of these activities, it focuses on developing its members, allowing them to apply the knowledge they have acquired to their work. A total of 24 volunteers were trained and subsequently provided with educational materials and funding to carry out health and nutrition education sessions for a total of 12 months, from May to October in both 2018 and 2019. The table presented in Table 1, outlines the progress of the activities over the 3-year project and the participation rate of children under the age of five and their caregivers in health/nutritional education.

**Table 1:**
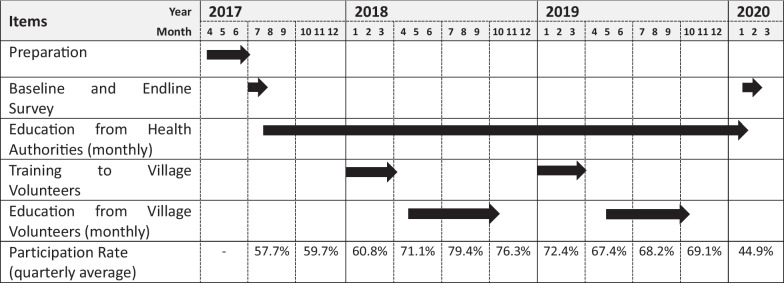
Implementation schedule of activities

Based on evidence that malnutrition starts during fetal development and increases during 6–23 months, which is the period of complementary feeding [15], both actors provided health/nutrition education in line with the concept of the “first 1000 days [16].” The education covered

1. Diversity of food during pregnancy,
2. Exclusive breastfeeding from 0 to 5 months,
3. Continuation of breastfeeding up to 23 months, and
4. Dietary diversity during complementary feeding from 6 to 23 months.

**Fig. 1:**
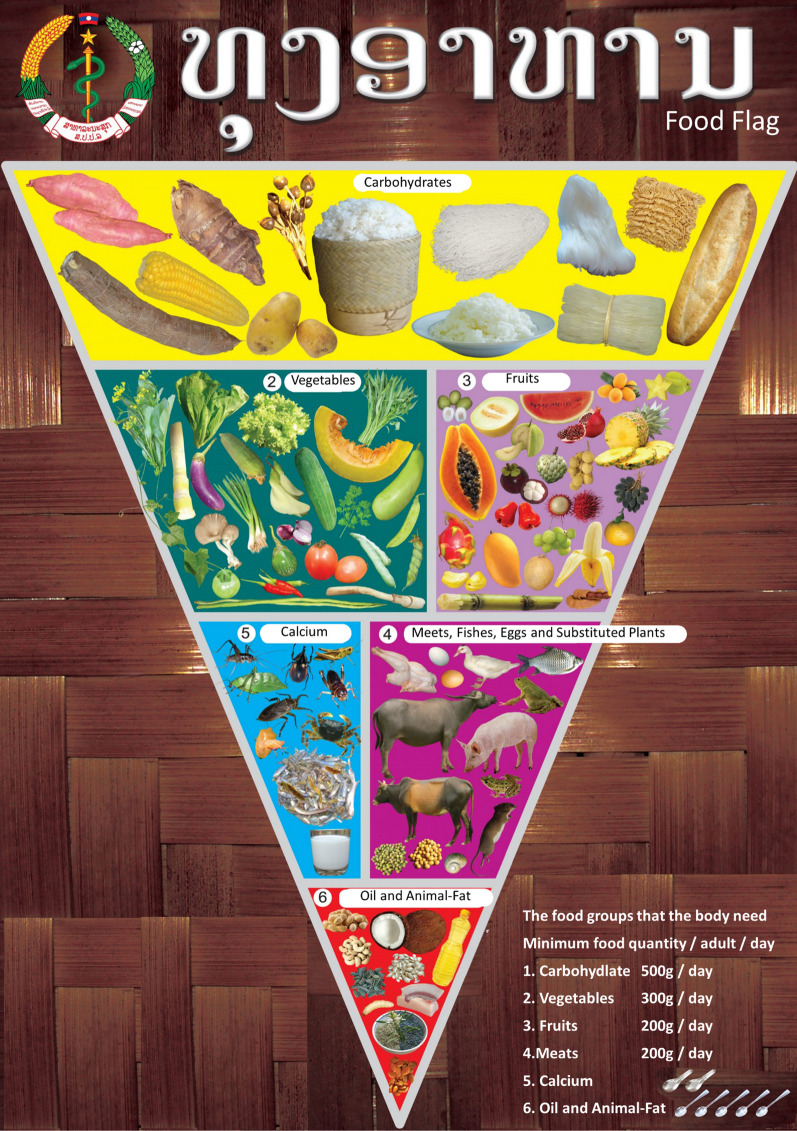
Material of health/nutrition education (dietary diversity) by ministry of health Lao PDR

Health education materials used the “Food Flag (Fig. 1)” issued by the Lao Ministry of Health to explain food groups and emphasize the importance of dietary diversity. Although the health/nutrition education provided by healthcare practitioners and community volunteers was generally similar in content, there were some differences. The education conducted by healthcare practitioners included explanations about the influence of local food taboos [17] and also addressed topics related to hygiene and disease prevention. On the other hand, the education provided by community volunteers should not have covered these topics. Instead, the volunteers provided a cooking demonstration founded by the project of complementary food (rice porridge) that incorporated various food groups. During the cooking demonstration, we actively emphasized utilizing locally accessible and cost-effective sources of high nutritional value, such as “eggs” and “cooking oil,” and vegetables and protein.

To encourage participation from mothers with targeted children, who were the target audience, in monthly outreach activities, the date of the events was communicated to the villages 1 day in advance. The village head utilized the village’s speaker system to promote the events to the target audience on the day before and early morning of the activity day. These outreach activities were held in communal spaces in the village, such as the village meeting hall or available space in a temple. Participants were provided with maternal and child health services, including health/nutrition education from healthcare practitioners. In addition, community volunteers conducted educational and awareness activities related to food and nutrition, including cooking demonstrations.

### Study design

Theis study design is a non-blinded, non-randomized single-group pre–post comparison trial within a quasi-experimental design. The project’s implementation allowed for a comparison of changes in IDDS between the baseline and endline surveys.

### Targets and data collection for evaluation

The survey included all children aged 6–59 months and their households residing in the target areas of Pharkhong, Phakouay-thong, and Phakouay-dong village. Enumerators, who were staff members affiliated with Xaybouathong District Health Office or the district hospital, used structured questionnaires for face-to-face interviews during home visits. Prior to the survey, enumerators outside the target areas underwent training to ensure uniformity in their survey abilities. The baseline survey was conducted in July 2017 (rainy seasons), and the endline survey was conducted in February 2020 (dry seasons).

During the interview, we covered the target population’s socio-demographic information, child’s diet based on a 24-h recall method, and questions related to the child’s health status and any special occasions from the previous day. We interviewed parents or caregivers who live with the children and are aware of their dietary intake and the ingredients used for cooking. To ensure accuracy, we followed the FAO’s dietary diversity guideline [7] and asked about all meals and snacks consumed by the child in a previous day. We categorized the participants based on their age into two groups, 6–23 months and 24–59 months, as dietary patterns evolve with age.

As the children residing in the target areas participated in the growth monitoring during the monthly outreach activities, height and weight measurements were not taken during the survey. Instead, the most recent values obtained during the activities were recorded. For height and weight measurements, healthcare practitioners trained in the WHO’s Child Growth Standard measurement techniques [18] conducted the measurements.

The survey questionnaires were developed through consultations with local healthcare practitioners, referring to Lao national data [10]. Before the survey, a pretest was conducted in villages outside the target areas to confirm no unintended variations in responses and to finalize the questionnaire content. After the survey, the data were entered into Microsoft Excel, and a double-check was performed to ensure accuracy and correct any errors in the input data.

### Data analysis

Based on the responses obtained from target children aged 6–59 months, the following exclusion criteria were applied for data analysis:

1. Parents or caregiver who were not aware of the target child’s dietary intake on the previous day
2. Participants with missing data on dietary information
3. Participants whose dietary intake differed significantly from the usual due to poor health on the previous day
4. Participants who had moved from other areas and had been residing in the area for less than 1 month (endline survey only)

For 6–59 month children who were included in the analysis, their sociodemographic characteristics were summarized using descriptive statistics. As an outcome indicator of this study, dietary diversity was scored based on the information obtained through the 24-h recall. Following the FAO report [7], the consumed food items were listed and classified into 16 food groups as the original units. Then, the count of consumption for each of the 16 food groups was merged to calculate the score for the 8 food groups of the Individual Dietary Diversity Score (IDDS) [19]: (1) grains, roots or tubers, (2) vitamin A-rich plants and foods, (3) other fruits and vegetables, (4) meat, poultry, fish and seafood, (5) eggs, (6) pulses, legumes and nuts, (7) milk and milk products, and (8) foods cooked in oil/fat. Furthermore, as providing supplementary information to assess the residents’ economic food access, the consumption of ‘snacks and soft drinks’, which required cash purchases, was also counted as an additional food group (Table 2).

**Table 2:** Food groups used for dietary diversity assessment and integration method with IDDS

16 Food Groups		Individual Dietary Diversity Scores (IDDS)	
1	Cereals	1	Grains, Roots and Tubers (16FG: 1,2)
2	White Roots and Tubers	2	Vitamin A-Rich Plants and Foods (16FG: 3,4,6)
3	Vitamin A Rich Vegetables and Tubers	3	Other Fruits and Vegetables (16FG: 5,7)
4	Dark Green Leafy Vegetables	4	Meat, Poultry, Fish and Seafood (16FG: 8,9,11)
5	Other Vegetables	5	Eggs (16FG: 10)
6	Vitamin A Rich Fruits	6	Pulses, Legumes and Nuts (16FG: 12)
7	Other Fruits	7	Milk and Milk Products (16FG: 13)
8	Organ Meat	8	Foods Cooked in Oil/Fat (16FG: 14)
9	Flesh Meats	Add	Snacks and Soft Drinks (16FG: 15,16)
10	Eggs		
11	Fish and Seafood		
12	Legumes, Nuts and Seeds		
13	Milk and Milk Products		
14	Oils and Fats		
15	Sweets		
16	Spices, Condiments, Beverages		

Height-weight were used, along with information on gender and date of birth, to calculate Height for Age (HAZ) and Weight for Height (WHZ) using the “Anthro” package in R. Z-scores below -2 were defined as stunting or wasting, respectively, according to the World Health Organization standards [2]. Weight for Age (WAZ) was not used as an evaluation indicator due to its susceptibility to the influence of height.

In our statistical analysis, we first utilized descriptive statistics to present the sociodemographic and economic characteristics of the target population. This involved reporting percentages, means, and standard deviations. To investigate differences between the baseline and endline data, we conducted bivariate non-parametric tests. Specifically, we employed the Mann–Whitney *U* test for continuous variables, and the Chi-square test or Fisher’s exact test for categorical variables.

Changes in IDDS were assessed using two methods: (1) the Chi-squared test or Fisher’s test for changes within each food group of IDDS, and (2) a generalized linear model, assuming that the probability distribution of IDDS, as the objective variable, follows a Poisson regression. The explanatory variables included baseline and endline surveys to indicate the intervention effect, as well as sociodemographic and economic factors of the children as covariates in the model. Model fit was evaluated using Akaike’s information criterion. We established a confidence interval of 95%, and statistical significance was determined at a significance level of 0.05. Some of the children were part of the same households, prompting an examination of potential household-level influences. However, given that approximately 80% of the subjects were from distinct households, this study concluded that household-level factors were not deemed impactful on the outcomes. Consequently, all variables were treated as individual characteristics in the data analysis. All data were entered into Microsoft Excel and analyzed using the statistical software R [20].

This study, while encompassing all children in the target area, is not influenced by selection bias. However, due to the unavailability of control group data at the beginning of the project, the interpretation of results must be approached with caution as this study relies on a single-group pre–post-comparison.

## Results

In the baseline survey, 258 children 6–59 months were targeted from the village registration and reached all. Following the exclusion criteria, 210 participants (81.4%) were extracted as eligible subjects who could be appropriately interviewed and from whom analyzable information was obtained. The same procedure was applied in the endline survey, where out of the 233 target participants, 205 (88.0%) were included in the analysis.

### Sociodemographic and anthropometric characteristics

Table 3 summarizes the sociodemographic characteristics of the study participants. The characteristics were compared between the baseline and endline surveys. No significant differences were observed in the participants’ sociodemographic and backgrounds in both surveys. As for economic status, last month electricity bill was slightly decreased from baseline but other indicators such as ownership of household assets were no difference was detected.

**Table 3:** Sociodemographic, economic and anthropometric characteristics between baseline and endline surveys

Items	Baseline2017 (*n* = 210)	Endline2020 (*n* = 205)	*P value*
Respondents*			
Mother	190 (90.5)	182 (88.8)	***0.009***^***b***^
Father	10 ( 4.8)	2 ( 1.0)	
Grandparents/Others	10 ( 4.8)	21 (10.2)	
Age groups*			
6–23 months	75 (35.7)	59 (28.8)	*0.160*^*b*^
24–59 months	135 (64.3)	146 (71.2)	
Age in Days^a^			
6–23 months	492 (159.7)	493 (154.7)	*0.969*^*c*^
24–59 months	1287 (299.1)	1322 (302.2)	*0.393*^*c*^
Gender*			
Male	101 (48.1)	95 (46.3)	*0.795*^*b*^
Female	109 (51.9)	110 (53.7)	
Villages*			
Phakouay-dong	47 (22.4)	52 (25.4)	*0.297*^*b*^
Phakouay-thong	80 (38.1)	87 (42.4)	
Phakhong	83 (39.5)	66 (32.2)	
Household size*			
2–4	73 (34.8)	55 (26.8)	*0.143*^*b*^
5–7	103 (49.0)	107 (52.2)	
8 ≤	33 (15.7)	43 (21.0)	
N/A	1 (0.5)	0 ( 0.0)	
Ownership of Household Assets*			
TV	150 (71.4)	160 (78.0)	*0.150*^*b*^
Mobile Phone	176 (83.8)	177 (86.3)	*0.558*^*b*^
Refrigerator	95 (45.2)	103 (50.2)	*0.356*^*b*^
Tractor	134 (63.8)	140 (68.3)	*0.390*^*b*^
Household Expenditure^a^			
Electricity bill (last month)^d^	35,451 (39,529)	29,598 (37,468)	***0.015***^***c***^
Child Health Status Yesterday*			
Healthy	210 (100)	187 (91.2)	**< *0.001***^***b***^
Unhealthy	0 (0.0)	18 (8.8)	
Stunting (HAZ)*			
− 2 <	128 (61.0)	119 (58.0)	*0.166*^*b*^
− 2 ≤ HAZ ≤ − 3	64 (30.5)	59 (28.8)	
− 3 >	15 (7.1)	16 (7.8)	
N/A	3 (1.4)	11 (5.4)	
Wasting / Overweight (WHZ)*			
+ 2 ≤	0 (0.0)	0 (0.0)	*0.150*^*b*^
− 2 < WHZ < + 2	186 (88.6)	181 (88.3)	
− 2 > WHZ ≥ − 3	21 (10.0)	12 (5.9)	
− 3 >	0 (0.0)	1 (0.5)	
N/A	3 (1.4)	11 (5.4)	

Bold indicates that, as a result of statistical analysis, the *p*-value has become 0.05 or lower

*Number (percentage: %)

^a^Mean (Standard Division)

^b^Chi-square test or Fisher’s exact test (applied when expected frequencies are less than five) for categorical variables

^c^Mann–Whitney *U* test for continuous variables

^d^The currency used is LAK, the Laotian Kip

The endline survey showed a significant increase in the number of children with poor health conditions on the previous day. However, significant changes in the prevalence of stunting and wasting were not detected. Infants aged 0–5 months were excluded from the study, and the observed prevalence rates for these conditions were relatively higher than the national average. In addition, there were no overweight children identified in either the 2017 or 2020 data.

### IDDS and ingredients

Theoretically, IDDS ranges from a minimum of 0 to a maximum of 8; however, in the observed data, the minimum score was 1, and the maximum was 7. Table 4 summarizes the changes in IDDS score (mean and total), scoring categories, and the proportion of children consuming each food group by age group from 2017 to 2020.

**Table 4:** Bivariate analysis of IDDS at baseline and endline: a two-age group comparison

Items	6–23 months		*P valu*e	24–59 months		*P valu*e
	2017 (*n* = 75)	2020 (*n* = 59)		2017 (*n* = 135)	2020 (*n* = 146)	
IDDS (total score)*						
1	1 (1.3)	3 ( 5.1)	**< *0.001***^**b**^	1 ( 0.7)	0 ( 0.0)	**< *0.001***^**b**^
2	13 (17.3)	1 ( 1.7)		17 (12.6)	6 ( 4.1)	
3	33 (44.0)	10 (16.9)		73 (54.1)	59 (40.4)	
4	17 (22.7)	19 (32.2)		31 (23.0)	45 (30.8)	
5	8 (10.7)	19 (32.2)		12 ( 8.9)	26 (17.8)	
6	3 (4.0)	6 (10.2)		1 ( 0.7)	10 ( 6.8)	
7	0 (0.0)	1 (1.7)		0 ( 0.0)	0 ( 0.0)	
8	0 (0.0)	0 (0.0)		0 ( 0.0)	0 ( 0.0)	
IDDS (mean)^a^	3.36 (1.05)	4.22 (1.24)	**< *0.001***^**c**^	3.29 (0.85)	3.83 (1.00)	**< *0.001***^**c**^
For each Food Group*						
1. Grains, Roots and Tubers	75 (100)	56 (94.9)	*0.779*^b^	134 (99.3)	146 (100)	*0.480*^b^
2. Vitamin A Rich Plant Foods	6 (8.0)	11 (18.6)	*0.074*^b^	29 (21.5)	43 (29.5)	*0.136*^b^
3. Other Fruits and Vegetables	35 (46.7)	45 (76.3)	**< *0.001***^**b**^	107 (79.3)	137 (93.8)	**< *0.001***^**b**^
4. Meat, Poultry, Fish and Seafood	61 (81.3)	53 (89.8)	*0.224*^b^	125 (92.6)	135 (92.5)	*1.000*^b^
5. Eggs	17 (22.7)	25 (42.4)	***0.024***^**b**^	26 (19.3)	65 (44.5)	**< *0.001***^**b**^
6. Pulses, Legumes and Nuts	4 ( 5.3)	8 (13.6)	*0.100*^b^	10 (7.4)	30 (20.5)	***0.002***^**b**^
7. Milk and Milk Products	43 (57.3)	50 (84.7)	**< *0.001***^**b**^	3 (2.2)	6 ( 4.1)	*0.504*^b^
8. Foods Cooked in Oil/Fat	20 (26.7)	11 (18.6)	*0.308*^b^	37 (27.4)	32 (21.9)	*0.332*^b^
A. Snacks and Soft drinks	41 (54.7)	30 (50.8)	*0.723*^b^	68 (50.4)	104 (71.2)	**< *0.001***^**b**^

Bold indicates that, as a result of statistical analysis, the *p*-value has become 0.05 or lower

*Number (percentage: %)

^a^Mean (Standard Division)

^b^Chi-square test or Fisher’s exact test (when the expected frequencies include zero) for categorical variables

^c^Mann–Whitney *U* test for continuous variables

The results showed that the 6–23 month and 24–59 month age groups significantly increased IDDS scores from 2017 to 2020. For the 6–23 month age group, the mean score increased from 3.36 to 4.22, with the median score increasing from 3 to 4 points. For the 24–59 month age group, the mean score increased from 3.29 to 3.83, with the median score increasing from 3 to 4 points.

The results of the multivariate analysis, adjusted for sociodemographic and economic factors of the children, also indicated statistically significant improvements in IDDS before and after the project (Table 5).

**Table 5:** Results of the generalized linear models with the IDDS of baseline and endline

Items	Total number of children (*n* = 415)			
	Estimate	SE	95% CI	*P* value
Intercept	− 117.1	34.9	[− 185.6, − 48.7]	**< *0.001***
Survey				
Baseline (2017)	Reference	–	–	–
Endline (2020)	0.059	0.017	[0.025, 0.093]	**< *0.001***
Villages				
Phakouay-Dong	Reference	–	–	–
Phakouay-Thong	0.146	0.068	[0.013, 0.281]	***0.032***
Phakhong	0.123	0.070	[− 0.014, 0.261]	0.080
AIC	1419.6			

Bold indicates that, as a result of statistical analysis, the *p*-value has become 0.05 or lower

SE Standard Error; CI Confidential Interval; AIC Akaike’s Information Criterion

Through AIC stepwise, “Age Groups”, “Gender”, “Child Health Status”, “Household Size” and “Ownership of Household Assets” were excluded from the model

Looking at the intake of each food group (Table 4), “Other Fruits and Vegetables” and “Eggs” significantly increased in both age groups. “Milk and Milk Products” showed a significant increase only in the 6–23 month age group, while “Pulses, Legumes and Nuts” and “Snacks and Soft Drinks” showed a significant increase only in the 24–59 month age group. There was no significant decrease in the consumption of any food group.

When examining the consumption patterns of each food group, “Grains, Roots or Tubers,” “Other Fruits and Vegetables,” and “Meat, Poultry, Fish and Seafood” were consumed by nearly 90% of the children. On the other hand, “Vitamin A-rich Plants and Foods,” “Pulses, Legumes and Nuts,” and “Foods Cooked in Oli/Fat” were consumed by less than 20% of the children. “Milk and Milk Products” showed an interesting pattern, with 60–80% of the children in the 6–23 month age group consuming it, but most children in the 24–59 month age group did not.

Furthermore, we listed the top five frequently consumed food items within each food group to understand the composition of these groups (Table 6). In the Roots and Grains group, glutinous rice remained the top choice in 2017 and 2020, and instant noodles ranked second. In the Vitamin A-rich group, we observed some differences between 2017 and 2020, with chayote shoots, tamarind, and pumpkin sprouts ranking high in 2017, while water spinach, pumpkin, and pumpkin sprouts took the lead in 2020. In the Other Fruits and Vegetables group, the top five items in 2017 were bamboo shoots, basil, leeks, cucumbers, and bananas, while in 2020, the order changed to leeks, coriander, onions, tomatoes, and basil.

**Table 6:** Comparing the top 5 consumed ingredients in each food group at baseline and endline

Items	Baseline 2017	Endline 2020
	6–59 months (*n* = 210)	6–59 months (*n* = 205)
1. Grains, roots or tubers	Glutinous Rice Instant Noodle Corns Rice Rice noodles	Glutinous Rice Instant Noodle Rice Corns –
2. Vitamin A-rich plants and foods	Gourd sponge young leaves Tamarind young leaves Pumpkin young leaves Morning glory Pumpkin	Pumpkin young leaves Morning glory Pumpkin Tamarind young leaves Green amaranth
3. Other fruits and vegetables	Bamboo shoot Basil Scallions Cucumber Banana	Scallions Corianders Onion Tomato Basil
4. Meat, poultry, fish and seafood	Small fishes Frog Catfish Pork Chicken	Small fishes Catfish Buffalo’s meat Chicken Pork
5. Eggs	Hen Egg Duck Egg – – –	Hen Egg Duck Egg – – –
6. Pulses, legumes and nuts	Soymilk Yard long bean – – –	Soymilk Yard long bean Pigeon Pea – –
7. Milk and milk products	Breastmilk Formula Milk Cow Milk (UHT) – –	Breastmilk Formula Milk Cow milk (UHT) – –
8. Foods cooked in oli/fat	Fried Egg Fried Fish Stir-fried Bamboo shoot Stir-fried Pork Stir-fried Locust	Fried Egg Fried Fish Stir-fried Pork Instant noodle soup Fried cricket
A. Snacks and soft drinks	Snacks Soft drinks Ice Cream – –	Snacks Soft drinks Smoothie Ice cream Condense milk (with water)

1 This table presents the top 5 observed food items within each food group, obtained from 24-h dietary recall information. If fewer than 5 food items were identified, it is indicated with ‘–’

2 The names of ingredients provided in the Lao language were translated into English with the assistance of local healthcare professionals. However, a thorough taxonomic examination to verify the accuracy of botanical names has not been conducted

Small fishes remained the top choice in both years in the Meat and Fish group, but the other ranked items varied. For Eggs, chicken and duck eggs were the only ones used. In the Legumes and Nuts group, soy milk ranked first in both surveys, followed by yard-long beans, with chickpeas appearing only in 2020. In the Milk group, breast milk ranked first in both years, followed by formula milk and cow’s milk.

We counted menu items that utilized oils and fats for the Oils and Fats group. Fried eggs/omelets took the top spot, and fried fish came in second place as the most popular choice. Unfortunately, we could not analyze the specific types of Snacks and Soft Drinks as there were no records of the snack and soft drink names.

Finally, we classified the IDDS into three levels and listed the food groups that were consumed by more than 50% in each group (Table 7). As a result, 217 participants (138 in 2017 and 79 in 2020) were categorized in the low diversity group with an IDDS score of 3 or below, and it was found that “Grains, Roots or Tubers,” “Other Fruits and Vegetables,” and “Meat, Poultry, Fish and Seafood” were more commonly consumed in this group. The moderate dietary diversity group, IDDS score of 4–5, consisted of 177 participants (68 in 2017 and 109 in 2020) and added “Eggs” or “Foods Cooked in Oil/Fat” as a more common food compared to the group before. The high dietary diversity group, comprising 21 participants (4 in 2017 and 14 in 2020), showed a trend of higher consumption of “Pulses, Legumes and Nuts,” “Milk and Milk Products.”

**Table 7:** Food groups with higher consumption by dietary diversity levels

	Baseline 2017	Endline 2020
	6–59 months (*n* = 210)	6–59 months (*n* = 205)
Low Dietary Diversity Group^a^	1. Grains, Roots and Tubers	1. Grains, Roots and Tubers
– 3 points or below –	3. Other Fruits and Vegetables	3. Other Fruits and Vegetables
	4. Meat, Poultry, Fish and Seafood	4. Meat, Poultry, Fish and Seafood
		A. Snacks and Soft drinks
Moderate Dietary Diversity Group^b^	1. Grains, Roots and Tubers	1. Grains, Roots and Tubers
– 4 and 5 points –	3. Other Fruits and Vegetables	3. Other Fruits and Vegetables
	4. Meat, Poultry, Fish and Seafood	4. Meat, Poultry, Fish and Seafood
	8. Foods Cooked in Oli/Fat	5. Eggs
	A. Snacks and Soft drinks	A. Snacks and Soft drinks
High Dietary Diversity Group^c^	1. Grains, Roots and Tubers	1. Grains, Roots and Tubers
– 6 points or above –	3. Other Fruits and Vegetables	3. Other Fruits and Vegetables
	4. Meat, Poultry, Fish and Seafood	4. Meat, Poultry, Fish and Seafood
	5. Eggs	5. Eggs
	8. Foods Cooked in Oli/Fat	6. Pulses, Legumes and Nuts
	A. Snacks and Soft drinks	7. Milk and Milk Products
		8. Foods Cooked in Oli/Fat
		A. Snacks and Soft drinks

This table compares food groups consumed by each of *Three Levels* of dietary diversity, with each group representing more than 50% consumption at baseline and endline

^a^Low Dietary Diversity Group was identified 138 children in 2017 and 79 children in 2020

^b^Moderate Dietary Diversity Group was identified 68 children in 2017 and 109 children in 2020

^c^High Dietary Diversity Group was identified 4 children in 2017 and 17 children in 2020

“Vitamin A-Rich Plants and Foods” consumption in all groups was below 50%. On the other hand, it became evident that “snacks and soft drinks” were consumed by 50% or more of the participants in many groups, even in low dietary diversity group in 2020.

## Discussion

In previous studies, individual and intensive interventions such as home visits have suggested their effectiveness [21]. In our study, we conducted a total of 30 health/nutritional education sessions by healthcare professionals and 12 sessions by trained volunteers, both of which were delivered to groups in community rather than individuals. However, the improvement in dietary content observed in this study is assumed to indicate a certain level of effectiveness of the intervention. This is supported by reports from various regions worldwide on the positive impact of community-based nutritional education and cooking demonstrations on addressing child malnutrition, especially in communities [22–24]. From this perspective, our research has yielded a significant outcome, demonstrating how nutritional education interventions can enhance children’s dietary diversity within the constraints of the rural Laotian environment.

### The changes children’s dietary diversity

The consumption of “Other Fruits and Vegetables” improved significantly in both age groups, increasing from 46.7% to 76.3% and from 79.3% to 93.8%, respectively, in this study. Methods to ensure dietary diversity through agricultural integration approach are widely recognized [25]. In Cambodia, it has been reported that the integration of agriculture and nutrition education led to improved consumption of “Other Vegetables and Fruits” [26]. While this study did not include agricultural activities, the significant improvement in consumption can be attributed to the access that residents have to crops they can cultivate and wild harvest, such as bamboo shoots. In rural areas of Lao, the utilization and sale of wild foods are common practices [27], and the food group can play a part in ensuring food security. However, there were differences in the food items consumed; in 2017, bamboo shoots and basil were the most consumed, while in 2020, onions, coriander, and scallions were more frequently consumed. These differences may be attributed to the change in available or easily accessible foods between the rainy season in 2017 and the dry season in 2020, considering that Xaybouthong District belongs to the lowland to midland areas, and crop cultivation (home gardens) can be difficult during the rainy season due to flooding.

The consumption of “eggs” also showed a substantial improvement, increasing from 22.7% to 42.4% and 19.3% to 44.5%, respectively. Eggs, known for their high nutritional value [28] and wide availability even in rural areas of Lao, were highlighted by volunteers in health/nutritional education as well as cooking demonstrations. While only chicken and duck eggs were identified, these were easily accessible when households managed poultry, providing a straightforward source. Even without livestock, eggs were available in local stores within each village, making access convenient. Thus, because the eggs had fewer barriers to access, it is likely that the behavior change was due to enhanced motivation among the population. Of course, as reported in other studies [29], economic factors (e.g., increased purchasing power) are a powerful driver for improving dietary diversity in LMICs. Therefore, it is essential to consider the possibility that changes in the economic status of residents may have affected this result. However, as shown in Table 3, the economic status of households to which the target children belong did not undergo dramatic improvement between the baseline and endline. Given this situation, it can be inferred that one of the factors contributing to the change in egg consumption is the influence of this project.

Regarding “Pulses, Legumes and Nuts,” a significant improvement was observed only in the 24–59 month age group, increasing from 7.4% to 20.5%. It was found that the increased consumption of soy milk, which is the most consumed food in this category, maybe due to an increase in children purchasing and consuming soy milk from nearby shops. As for “Milk and Milk Products,” a significant improvement was observed only in the 6–23 month age group, increasing from 57.3% to 84.7%. This improvement can be attributed to the influence of health/nutrition education promoting continued exclusive breastfeed and breastfeeding up to the age of 2 years. Cow’s milk consumption was observed in both 2017 and 2020, but the numbers were 3 in 2017 and 2 in 2020, indicating that cow’s milk (Ultra-High Temperature) from Thailand is available in that areas but is rarely consumed by children.

Finally, similar to reports in Cambodia [30, 31], high consumption of unhealthy ‘Snacks and Soft Drinks’ among children was observed in rural Lao. Over half of children aged 6–23 months were found to partake in such consumption, with a significant increase noted among children aged 24–59 months, ranging from 50.4% to 71.2%. In Nepal, it has been reported that Unhealthy snack food and beverage account for 24% of children’s calorie intake [32], and these unhealthy foods are associated with deteriorating dietary quality among children [33]. Such practices could potentially exacerbate malnutrition in infants and increase the risk of future non-communicable diseases [1]. Moreover, over half of the children consumed these items, even in the group with low dietary diversity (3 points or below). This suggests that low dietary diversity does not necessarily indicate economic insecurity in food procurement, but rather other factors, such as the ‘child’s pester power,’ might influence how parents allocate their money and incentivize the purchase of it [34] [35]. In this study, health/nutritional education focused on foods that should be consumed from the perspective of dietary diversity. However, it is desirable to include education on foods that should not be given to children.

### The un-changes children’s dietary diversity

Four food groups showed no significant change, and two were “Grains, Roots and Tuber” and “Meat, Poultry, Fish and Seafood” These two food groups had high consumption rates in 2017 and continued to be prominently consumed in 2020. This can be attributed to glutinous rice and protein sources being essential components of people’s diets in Lao, even in rural, serving as the ‘staple’ and ‘main dish.’ As expected, the high consumption of home-grown glutinous rice is not surprising, but it is interesting to note that instant noodles ranked second. This indicates that even for items that need to be purchased, the local food practices involve acquiring them when necessary. Instant noodles, in particular, have been reported in other studies to the main resource of salt intake [36], and its convenience influencing purchasing behavior. While it is essential to promote moderation in the consumption of these unhealthy foods, it is important to acknowledge that foods such as instant noodles are chosen for their strong convenience factor. Therefore, in addition to encouraging reduced consumption, exploring methods such as using nutrient-fortified instant noodles might also be considered [37]. Protein sources for main dishes are frequently sourced from hunting and gathering, with small fish, frogs, and catfishes being commonly consumed. Chicken, pork, and buffalo meat were also confirmed, suggesting that even purchased items are regularly on dining tables.

Another food item that showed no significant change was “Foods Cooked in Oli/Fat.” In the context of our health/nutrition education and cooking demonstrations, we actively advocated for incorporating cooking oil. However, the outcomes were different from eggs. It is a recognized fact that energy intake derived from oil/fats is relatively modest within the food customs of Lao [38], which might be intricately linked to prevailing eating habits. Despite cooking oils being accessible through local stores, as portrayed in Table 7, it indicates a discernible utilization pattern within specific recipes. These inclinations, likely shaped by parental preferences [39] and other culinary dynamics, underscore the need for a more profound exploration to unravel the underlying intricacies of households’ dietary and procure behaviors.

The last food group that showed no significant change was “Vitamin A-Rich Plants and Foods,” encompassing dark green leafy vegetables, yellow and orange root vegetables, and fruits [8]. Despite baseline and endline measurements revealing consumption levels below 30%, a closer examination of the ingredients, such as pumpkin (fruit and leaves) and morning growly, suggests that these are relatively accessible even in rural areas. The background of such results lies not only in children’s food preferences but also in the fact that vegetables rich in “Vitamin A-Rich Plants and Foods” such as pumpkin are cultivated foods rather than wild plants. Nutritional education and agricultural interventions, such as those in Cambodia [26], have been successful in improving the consumption of this food groups. Hence, factors such as the need for land and resources for cultivation, as well as the availability of cultivation techniques, are believed to influence this outcome. Furthermore, it is plausible that the lack of precise motivation could have stemmed from the categorization of these food items as part of the “vegetable” or “fruit” in health/nutrition education (Fig. 1). This contributed to the diminished effectiveness of motivational strategies.

### Association between child dietary diversity and anthropometric status

Previous research has shown that dietary diversity effectively improves child malnutrition [4, 6]. In this study, although significant improvements in dietary diversity were observed, there were no statistically significant changes in the prevalence of stunting and wasting. Three factors may explain this finding. First, it is crucial to consider the timing of the health/nutrition education conducted after the third quarter of 2017 and the activities led by community volunteers that started after the second quarter of 2018. As the evaluated children were under 5 years, it may take some time for the effects to become apparent. As concluded in other studies [26, 40], it appears that time is necessary for the outcomes of improvements in infant and young child nutrition to become evident, which can also be interpreted from the results of our study.

Second, even if dietary diversity improves, the consumption of nutrients exceeds the improvement within the body. Sayasone [41] and Takakura [42] investigated the prevalence of parasitic infections in children in rural Lao and reported on the association between parasitic infections and malnutrition. Chronic parasitic infections may hinder the proper utilization of consumed nutrients for growth and development. Hence, it is necessary to investigate these effects further. Moreover, in response to these issues, Lao conducts biannual mass deworming as part of its Mother and Child Health Services. This aligns with UNICEF’s framework [43] and undoubtedly plays a role in ensuring the proper and effective delivery of the services, contributing to the improvement of malnutrition. However, it is crucial to consider the limitations of the supplemental approach. In this regard, it is important to take into account the findings by Baffour et al. [44], who reported that supplementing deficient micronutrients did not lead to a reduction in stunting.

The last factor is the impact of maternal malnutrition, specifically during the fetal period. Anemia in pregnant women in Lao has been reported to be over 40% [12], and malnutrition during the ‘First 1000 days [16]’ begins during pregnancy. Reports also indicate that the minimum dietary diversity indicator for pregnant women in rural areas (MDD-W) is around 30%, and there are known food taboos during breastfeeding [45]. Therefore, while studying children’s dietary diversity is essential, understanding the overall picture of a child’s food and nutrition is crucial for reducing stunting or wasting.

### Strengths and limitations

The study focused on only a part of the Xaybouathong district, and no control groups were available, because all targets had to benefit from the development cooperation project, which could lead to difficulty dividing intervention and other confounding effects from the results. However, the data were collected from approximately 90% of children 6–59 months residing in the target villages, and possible confounders could be included in statistical analysis as co-variables. Furthermore, this study is active research conducted by the principal investigator, who stayed in the target area for 3 years and interacted with local people to reveal their dietary diversity. The information on the diet was collected through interviews with well-informed local investigators, making the data highly reliable. Therefore, this study provides valuable insights into dietary diversity and its changes in rural Lao.

Except for that, there are three limitations to this study. First, it is challenging to quantify the exposure of mothers targeted in education. This limitation weakens the evidence for whether the observed changes in dietary diversity were genuinely caused by health/nutrition education. Nevertheless, the most crucial aspect of this study is identifying how to improve dietary diversity among children in future nutrition policies, making it highly valuable information. Second, the children’s dietary survey method relied on a single 24-h recall data. The weakness of a 24-h recall data is that daily diet varies, and it can be affected by day-to-day fluctuations. Therefore, it is essential to interpret the results of this study with these considerations in mind. Finally, there is a limitation related to seasonal effects. The study was conducted during the rainy season in 2017 and the dry season in 2020, which could have influenced the food procurement environment due to seasonal differences. However, it is generally reported that the rainy season tends to be abundant in natural food resources [46]. Therefore, the study’s finding of improved dietary diversity during the dry season can be evaluated as an improvement surpassing the adverse effects of the seasons.

## Conclusions

Improving child’s dietary diversity in a resource-limited situation is challenging. However, this study demonstrated that continuously implementing health/nutrition education in rural Lao PDR still has a vital role in delivering the necessary information to caregivers and can lead to a change in their food preparation practice [47].

One of the challenges of dietary diversity in LMICs is protein deficiency. However, in the study area, abundant natural resources allowed many children to consume meat and fish [48]. Understanding which food groups are deficient in dietary diversity can effectively improve their diet. The study showed that promoting the intake of eggs, legumes and nuts, and oils/fats was feasible for the residents. Using such strategies, it is possible to increase the frequency of consuming three, four, and five food groups and contribute to the nutritional improvement of children. Indeed, as it has been reported that food-based nutritional improvements alone may not adequately supplement certain micronutrients [49], it is essential to consider multisectoral approaches [50], such as agriculture approach and food fortification approach alongside nutritional education.

On the other hand, it became evident that even children with limited dietary diversity consumed more than half of their snacks and soft drinks. This suggests that caregivers are incentivized to provide these foods to children. Even when accessible foods are available, further investigation is needed to understand the underlying reasons for parents’ food choices.

Overall, this study provides valuable insights into improving dietary diversity and nutrition in resource-limited settings, emphasizing the importance of targeted interventions and understanding the factors influencing food choices in the community.

## Data Availability

The data supporting the findings of this study are available upon reasonable request to the corresponding author, YS. However, please note that the data cannot be publicly disclosed due to the inclusion of personal information that could compromise the privacy of the research participants.

## Abbreviations

FAO: Food and agriculture organization
HAZ: Height-for-age Z-scores
IDDS: Individual dietary diversity score
LMICs: Low-and-middle-income countries
Lao PDR: Lao People’s Democratic Republic
MDD: Minimum dietary diversity
MDD-W: Minimum dietary diversity for women
WAZ: Weight-for-age Z-scores
WHO: World Health Organization
WHZ: Weight-for-height Z-scores
UHT: Ultra-high temperature
